# Tuning in to recovery: influence of music on emotional well-being during mealtime in inpatient facilities for eating disorders

**DOI:** 10.1186/s40337-024-00970-9

**Published:** 2024-01-15

**Authors:** Paolo Meneguzzo, Elisa Baron, Silvia Marchesin, Anna Maria Andretta, Lisa Nalesso, Sonia Stella, Patrizia Todisco

**Affiliations:** 1 Department of Neuroscience, University of Padova, 35128 Padua, Italy; 2 Padova Neuroscience Center, University of Padova, Padua, Italy; 3 Eating Disorders Unit, Villa Margherita - Neomesia, Arcugnano, Vicenza Italy

**Keywords:** Mealtime, Eating disorders, Inpatient, Music, Positive emotions

## Abstract

**Background:**

In rehabilitating eating disorders (ED), mealtimes are critical but often induce stress, both for restrictive and binge-purge disorders. Although preliminary data indicate a positive effect of music during mealtime, few studies employ an experimental approach. This study examines the influence of background music during mealtime in an inpatient ward setting, offering a real-world perspective.

**Methods:**

Fifty-one women diagnosed with ED participated in this within-subjects study. Over two weeks, during lunch and dinner, they were exposed to three randomized music conditions: no music, focus piano music, and pop music. The self-report questionnaires captured affective states, noise levels, and hunger, while trained dietitians recorded food consumption and eating rituals.

**Results:**

The absence of music led to an increase in uneaten food (*p* = 0.001) and the presence of eating rituals (*p* = 0.012) during mealtimes. Significantly, only silence during mealtime reduced positive emotional states, while background music maintained positive emotions (*p* < 0.001). No specific differences emerged between the two types of music (focus piano and pop).

**Conclusions:**

These findings affirm the positive impact of background music during mealtime in real-world settings, enhancing the potential of inpatient eating rehabilitation programs for individuals with ED. More studies are needed to validate and extend these results, particularly in outpatient settings.

## Abstract (plain language summary)

Mealtimes can be stressful for people with eating disorders (ED). This study looked at how background music during meals could help. We had 51 women with ED in an inpatient ward. They ate lunch and dinner without music, calming piano music, or pop music for ten days. We asked them about their feelings, noise, and hunger. Dietitians noted what they ate and any rituals. We found that not having music led to more uneaten food and eating rituals. Surprisingly, complete silence reduced positive emotions. All types of music kept positive feelings, with no difference between them. Music helped to make mealtimes better for these patients. But more research is needed, especially for patients outside the hospital.

## Background

Eating disorders (ED) encompass a group of severe psychiatric conditions characterized by dysfunctional eating habits, significantly diminishing an individual's quality of life and often necessitating extended treatments [1–3]. From a transdiagnostic perspective, the psychopathological core of ED revolves around concerns related to body image and dysfunctional emotional regulation, involving behaviors such as binge eating or restraint [4–6]. In fact, weight and eating habits are central to the psychopathology of ED [7], with nutritional rehabilitation serving as a primary treatment goal across the entire spectrum of ED [8, 9].

Despite the crucial role of nutrition in ED treatment, empirical knowledge about meal support remains limited [10, 11], especially within inpatient settings [12], and the focus has often been on anorexia nervosa [13]. This could be attributed to differences in the rehabilitation of restrictive and binge-purge eating behaviors, which may have guided the research toward more extreme and unstable conditions [14, 15]. Patients undergoing ED treatment may have distinct objectives during mealtimes, influenced by the nature of their diagnosis and the specific challenges they face [16]. For example, individuals with anorexia nervosa may have goals centered around increasing food intake and addressing malnutrition, while those with bulimia nervosa may focus on establishing regular eating patterns and reducing binge-purge cycles [17–19]. Staff-supervised mealtimes in ED treatment serve a crucial purpose, functioning as pivotal moments for patients with diverse ED diagnoses and various types of eating pathology. These structured mealtimes aim to provide a supportive environment where patients can work towards their individualized goals, taking into consideration the nuances of their specific ED diagnosis. For example, dietitians manage the composition and administration of meals, with the support of other professionals such as nurses who can help improve the emotional climate, reduce rituals, minimize compensatory behaviors, and follow the goals established outside the dining area [18, 19].

Mealtime in an inpatient environment can be particularly challenging due to its associations with eating, changes in body weight, and the potential for negative affect, fear, and anxiety [20, 21]. Previous studies have suggested strategies to create comfortable, safe, and structured environments, encourage regular conversations, and provide opportunities for patients to address their difficulties [10, 22]. Other strategies may include avoiding negative feelings or providing comfort to patients [13]. Through experimental methods, the evaluation of diverse strategies, including vodcasts and music, has revealed that interventions aimed at helping individuals cope with anxiety and preventing mood decline can positively influence increased food consumption [23, 24]. However, due to the limited understanding of the best approaches to support patients, strategies can vary [12, 25], often leaving it to individual initiative and resulting in diverse approaches and outcomes.

Addressing these challenges requires an exploration of holistic and transdiagnostic approaches to eating disorders. This is particularly important due to the possible presence of individuals with different diagnoses in the same space during mealtime. Considering shared psychopathological elements, the intervention should focus on reducing restraint, addressing food rituals, challenging dysfunctional beliefs about food, and minimizing emotional activation triggered by food presentation and assumptions [10, 16]. Music, with its potential as a transdiagnostic intervention, has been gaining recognition in the treatment of various psychiatric conditions [26]. Music-based interventions have been recognized as effective in reducing stress responses [27] and decreasing state anxiety [28], while also modulating the autonomic nervous system [29]. Recent studies have suggested that music-based interventions can offer benefits that cut across diagnostic boundaries, helping individuals struggling with different subtypes of ED cope with emotional dysregulation, improve their overall psychological well-being, and reduce anxiety [30–32]. A qualitative study conducted after the meal indicates that music assisted individuals in diverting their attention from the recently consumed meal, taking a break from anxiety, and connecting with their peers. Indeed, music listening seems to increase mindfulness following a stressor [33]. However, there are several aspects that should be considered in the implementation of music in a specific intervention, from personal preferences to specific characteristics like rhythm or sounds. For example, it seems that a slow tempo might produce greater relaxation and less tension than faster tempo music [34–36], but evidence is still limited.

Therefore, this article aims to evaluate the influence of music, or its absence, during mealtime within a specialized inpatient service for ED. Additionally, it explores the potential of music as a facilitator for approaching food and for reducing negative affective effects, particularly within a transdiagnostic context. Notably, given the current lack of clear evidence regarding the specific type of music that may be beneficial, the study aims to contribute insights in this regard. Our primary hypothesis is twofold: first, we hypothesize that music during mealtime can help individuals with ED cope by producing positive effects on mood and decreasing anxiety, leading to better emotional responses than those experienced in silence; second, we hypothesize that music during mealtime will yield additional benefits, such as a stronger desire to eat, ultimately aiding participants during meals. The secondary objective is to evaluate the differences between focused/relaxation music and general music in terms of tolerability and other effects on meal rituals or food intake, to identify variations between specific types of music.

## Method

### Participants

This study employed a within-subjects experimental design. Fifty-one women with an ED were recruited for this study while receiving inpatient care at Casa di Cura Villa Margherita in Arcugnano, Vicenza, Italy, a specialized ward for psycho-nutritional rehabilitation [37]. Enrollment was based on presence in the inpatient facility and none of the patients refused to participate. The inclusion criteria mirrored the hospitalization requirements, including (a) an age range between 13 and 60 years and (b) the absence of severe psychiatric conditions such as schizophrenia or bipolar disorder, medical comorbidities, or neurological trauma or disorders. Trained psychiatrists diagnosed ED in all participants, following the criteria outlined in the DSM-5 [38].

All participants voluntarily consented to participate in the evaluation, and patients under 18 years of age required parental consent. No participant was remunerated for their involvement. This study adhered to the principles of the Declaration of Helsinki and its subsequent amendments and received approval from the Vicenza Ethics Committee.

### Questionnaires

Eating psychopathology within the sample was assessed using the Eating Disorder Examination Questionnaire (EDE-Q), a 28-item self-report questionnaire widely used to evaluate specific concerns [39]. It comprises four subscales, namely eating restraint, eating concern, shape concern, and weight concern, in addition to producing a global score.

The Positive and Negative Affect Schedule (PANAS) is a self-report questionnaire that features two 10-item scales designed to measure positive and negative affect [40]. Each item was rated on a 5-point Likert scale ranging from 1 (not at all) to 5 (very much).

To assess various subjective sensations, specific 10 cm visual analog scales were utilized to measure hunger, satiety, desire to eat, perceived stress, and mealtime difficulty. Participants were asked to indicate the intensity of each sensation by marking on the scales. Following the meal, the volume of the music and its pleasantness were evaluated using an 8-point Likert scale, ranging from 0 (absent) to 7 (very high/very pleasant).

For the evaluation of eating rituals, a streamlined checklist adapted from a previous study was used, which contained three elements: cutting food into small pieces, patting the food dry, and focusing on one food item at a time [41]. The presence or absence of rituals was determined for each meal, with scores ranging from 0 (no rituals) to 1 (rituals present in each meal). Dietitians were trained to standardize the assessment, and two evaluators reached a consensus on the presence or absence of rituals.

Finally, the ability to adhere to the correct mealtime, set at 20 min for each course, was assessed.

### Meal conditions and procedure

Over a span of four weeks, from April 2022 to July 2022, this study involved the randomization of three different background music conditions during lunch and dinner, occurring from Monday through Friday. For the first two weeks and the last two, the meal compositions remained consistent and identical for all participants. General meal planning details are provided in Table 1. Dietitians meticulously managed individual differences in meal compositions, recording these for subsequent comparisons with participants. The three background music conditions included no background music, continuous classical music featuring only a piano, termed 'focus music,' and a preset pop music playlist. The randomization, which included the number of meals and conditions, was performed using the Excel function (RAND). All other variables during mealtime remained constant, encompassing ward conditions, timing, meal planning, communal groups, and the presence of both a nurse and a dietitian. Participants were seated in groups of three or four at tables and were encouraged to engage in conversation under all conditions.

**Table 1:** Meals planning. Weeks 1 and 2 were repeated identically

Day	Meal	Food	Energy (kcal)	Proteins (grams)	Lipid (grams)	Carbohydrates (grams)	Conditions
*Weeks 1 and 2*							
Monday	Lunch	Crepes with ricotta cheese and spinach, tilapia with olives and capers, zucchini, bread	1035	65.8	52.6	77.1	Pm/Nm
	Dinner	Rice with oil, lentils, bread	779	17.9	21.4	122.3	Nm/Fm
Thursday	Lunch	Pasta with rag, turkey roast, salad, bread	931	47.4	40.3	102.2	Fm/Nm
	Dinner	Zucchini soup with toast bread, soufflé ricotta and spinach, bread	778	26.1	33.3	93.7	Pm/Fm
Wednesday	Lunch	Emmer with vegetables, meatballs, broccoli, bread	909	32.3	40.7	102.2	Nm/Pm
	Dinner	Pea soup, chicken breast with butter, salad and carrots, bread	688	34.4	41.1	42.7	Pm/Fm
Tuesday	Lunch	Rice with zucchini, ricotta, artichokes, bread	897	29.86	50.75	91.4	Nm/Pm
	Dinner	Pasta with oil, chickpeas, bread	768	27.2	25.3	118.6	Fm/Nm
Friday	Lunch	Gnocchi with tomato sauce, fish, spinach, bread	997	45.1	34.7	103.3	Pm/Nm
	Dinner	Pasta and beans, puff pastry filled with vegetables, bread	809	21.7	41.9	91.8	Fm/Pm
*Weeks 3 and 4*							
Monday	Lunch	Pasta with tomato sauce, hamburger, peppers, bread	1108	44.8	55.2	113.4	Pm/Nm
	Dinner	Vegetable soup with bread toast, turkey steak, spinach, bread	795	53.3	33.2	69.9	Fm/Pm
Thursday	Lunch	Barley with zucchini, lentils, bread	704	20.4	22.4	97.9	Nm/Fm
	Dinner	Rice with tomato sauce, cod, broccoli, bread	869	38.9	34.5	111.4	Pm/Pm
Wednesday	Lunch	Pasta with mozzarella and fresh tomato, pork roast, salad, bread	1115	40.3	63.2	101.8	Fm/Pm
	Dinner	Rice with vegetables, ham, zucchini, bread	848	30.6	37.3	102.5	Nm/Nm
Tuesday	Lunch	Lasagna with vegetables, chicken roast, green beans, bread	1018	52.3	42.1	109.6	Fm/Fm
	Dinner	Carrots soup with toast bread, asiago cheese, eggplant, bread	894	35.3	51.9	61.1	Nm/Pm
Friday	Lunch	Rice with fish, fish with tomato sauce, salad, bread	873	43.6	36.2	100.5	Pm/Nm
	Dinner	Soup with legumes and emmer, bresaola, cheese, and rocket salad, grilled vegetables, bread	871	49.5	35.8	84.3	Fm/Fm

Conditions reported the exposure to music for each meal for the first/second or third/fourth week

*Nm* no music, *Fm* focus music, *Pm* playlists music

General meal planning encompassed six distinct mealtimes, including breakfast, lunch, dinner and three snack sessions, each customized to address specific nutritional rehabilitation needs related to the participant's diagnosis. We focused our evaluation exclusively on lunch and dinner due to their lower interpersonal variability compared to other mealtimes. Additionally, both lunch and dinner were managed uniformly by dietitians in terms of food composition and quantity, ensuring consistency for all patients. The remaining daily meals exhibited variations according to the individual's diagnosis and unique nutritional requirements.

Throughout mealtime, dietitians meticulously recorded each participant's meal composition and the amount of uneaten food at the meal's conclusion. The remaining food was classified as nothing, a quarter, half, three-quarters, or all. All dietitians were trained equally for this evaluation before the study, worked together in the ED facility for years, and were supervised by the same dietitian. The nutritional and energy composition was evaluated according to national guidelines [42] in a separate moment using notes about the quantity of food left by each participant. This operation was necessary for comparing different meals during the weeks.

Noise levels were measured using a dedicated cell phone application called 'DecibelX' placed in the room's center, the device captured the mean noise level during the meal, with data reported in decibels. The goal was to evaluate the consistency of the music volume across different scenarios.

Different dietitians managed background music and meal planning during the specific study weeks. This randomization was planned to eliminate any potential associations between meal compositions and music conditions. Before each meal, information on hunger, satiety, the desire to eat, and PANAS scores was collected using a pencil-and-paper approach. After mealtime, participants reported psychological difficulty with meals, music volume, loudness, and provided information on hunger, satiety, the desire to eat, and PANAS. The post-meal questionnaires were filled out 5 min after the end of the meal. Additionally, during meals, a dietitian collected information about eating rituals, the ability to adhere to mealtimes, and details about what the participants consumed or left on their plates. During the course of the study, all participants participated in ten meals, being exposed to all conditions at least three times.

### Statistical analysis

We conducted a power analysis prior to recruitment based on similar studies with community volunteers, which indicated that a minimum of 25 subjects would be required to detect differences in energy intake with an α = 0.05 and a power of 80% [43]. Given the clinical condition of our sample, we opted to double the sample size.

All analyses necessitated a nonparametric approach due to the distribution of the data. The Kruskal–Wallis test was used to assess differences among diagnoses for demographic and clinical variables, as well as variations between different musical conditions. Pairwise comparisons were adjusted using the Bonferroni correction.

For each participant, mean responses were assessed both before and after at least three meals, and under different music conditions. A Box-Cox transformation was applied to the variables assessed before and after meals, including hunger, satiety, desire to eat, stress, and PANAS scores. This transformation enabled us to employ repeated measure ANOVA analyses, with pairwise comparisons adjusted using the Bonferroni correction. Additionally, the interaction between time (pre-post meal) and music conditions was assessed using repeated measures ANOVA.

A significance level was established for all analyses at *p* < 0.05. We conducted all data analyzes using IBM SPSS Statistics 25.0 software (SPSS, Chicago, IL, USA).

## Results

### Demographic characteristics

The included sample comprised 51 women: 31 had a diagnosis of anorexia nervosa, nine had a diagnosis of bulimia nervosa, five had a diagnosis of binge eating disorder, and six participants were diagnosed with other feeding and eating disorders. All the participants were white cisgender women. The sample was characterized by an average age of 25.22 ± 11.33 years and an average BMI of 20.34 ± 7.80 kg/m^2^. For clinical details, see Table 2.

**Table 2:** Demographic and clinical description of the participants

	Total sample N = 51	AN n = 31	BN n = 9	BED n = 5	OSFED n = 6
Age, years	25.22 (11.33)	22.94 (8.96)	22.33 (6.34)	31.40 (10.59)	24.50 (7.76)
BMI, kg/m^2^	20.34 (7.80)	16.89 (3.07)	20.80 (3.56)	40.50 (7.94)	20.69 (0.69)
EDE-Q Restraint	4.09 (1.42)	4.21 (1.35)	3.71 (1.58)	3.76 (1.87)	4.32 (1.32)
EDE-Q Eating concern	3.87 (1.09)	3.62 (1.08)	3.83 (1.14)	4.96 (0.82)	4.40 (0.51)
EDE-Q Shape concern	5.21 (0.91)	5.12 (0.79)	5.18 (1.52)	5.53 (0.38)	5.48 (0.70)
EDE-Q Weight concern	4.67 (1.17)	4.52 (1.07)	4.64 (1.72)	4.96 (0.88)	5.32 (0.79)
EDE-Q Global score	4.45 (0.92)	4.37 (0.85)	4.34 (1.28)	4.75 (0.94)	4.88 (0.63)

Means and standard deviations between brackets. AN: anorexia nervosa, BN: bulimia nervosa, BED: binge eating disorder, OSFED: other specified feeding and eating disorders

No differences emerged in the mean energy intake planned for participants (*H* = 2.250, *p* = 0.522) or the actual mean energy consumed (*H* = 0.569, *p* = 0.903) regarding the possible effects due only to the different diagnoses of ED (see Table 2 for details).

### Composition of meals

Table 3 compares the composition of the meal, the rituals, and the timing. Specific differences emerged for untaken energies (η_p_^2^ = 0.047), rituals (η_p_^2^ = 0.064), and pleasingness (η_p_^2^ = 0.612), with the condition without music that reported more negative results for all the features.

**Table 3:** Composition of meals and comparison between music conditions

	No music	Focus music	Playlist music	*H*	*p*	Post hoc
Meal energy planned (kcal)	873 (53)	850 (78)	879 (41)	4.815	0.090	
Eaten energy (kcal)	776 (114)	804 (124)	816 (121)	6.343	0.042	Nm < Pm (*p* = 0.041)
Uneaten energy (kcal)	97 (107)	45 (81)	63 (103)	14.138	0.001	Fm < Nm (*p* = 0.001) Pm < Nm (*p* = 0.010)
Eaten proteins (grams)	29.43 (10.39)	33.63 (9.82)	35.09 (6.64)	14.909	0.001	Nm < Fm (*p* = 0.019) Nm < Pm (*p* = 0.001)
Eaten lipid (grams)	35.89 (5.45)	36.11 (6.64)	38.22 (7.89)	8.187	0.017	Nm < Pm (*p* = 0.027)
Eaten carbohydrates (grams)	85.75 (18.32)	86.62 (16.18)	81.44 (14.97)	4.748	0.093	
Eating rituals	0.52 (.42)	0.28 (.35)	0.36 (.35)	8.869	0.012	Fm < Nm (*p* = 0.009)
In time	0.84 (.28)	0.85 (.26)	0.88 (.19)	0.174	0.917	
Mealtime Difficulty	5.47 (2.93)	5.34 (2.60)	5.62 (2.82)	0.125	0.882	
Volume of the music	0.06 (0.18)	1.76 (0.76)	3.16 (0.92)	122.927	< 0.001	Nm < Fm (*p* < 0.001) Nm < Pm (*p* < 0.001) Fm < Pm (*p* < 0.001)
Pleasingness	0.34 (1.15)	3.70 (1.75)	4.80 (1.61)	96.426	< 0.001	Nm < Fm (*p* < 0.001) Nm < Pm (*p* < 0.001)
Noise (dB)	50.20 (7.21)	54.70 (5.08)	57.36 (3.46)	8.757	0.013	Nm < Pm (*p* = 0.009)

Means and standard deviations between brackets

*Nm* no music, *Fm* focus music, *Pm* playlists music

### Within-subjects analyses

The means and standard deviations of the psychological evaluations in the three music conditions are reported in Table 4, with differentiation between pre-post mealtimes.

**Table 4:** Psychological evaluation before and after meals

		No music	Focus music	Playlist music	Time (pre-post meal) by music condition
Hunger	Pre	2.79 (2.44)	2.65 (2.23)	2.77 (2.51)	F = 0.189 *p* = 0.828
	Post	0.49 (1.02)	0.58 (1.13)	0.60 (1.26)	
Satiety	Pre	5.00 (2.92)	5.10 (2.76)	5.10 (2.87)	F = 0.129 *p* = 0.879
	Post	8.89 (1.78)	8.72 (1.94)	8.91 (1.69)	
Desire to eat	Pre	2.60 (2.49)	2.61 (2.45)	2.85 (2.79)	F = 0.411 *p* = 0.664
	Post	0.65 (1.28)	0.99 (1.85)	0.65 (1.28)	
Stress	Pre	6.36 (2.87)	6.00 (2.87)	6.20 (2.77)	F = 0.326 *p* = 0.722
	Post	6.20 (3.11)	6.23 (2.93)	6.20 (3.11)	
PANAS pos	Pre	19.51 (6.11)	18.13 (6.63)	19.88 (6.60)	F = 26.920 *p* < 0.001
	Post	14.63 (5.79)	19.51 (6.51)	19.44 (6.67)	
PANAS neg	Pre	26.67 (13.62)	25.33 (8.06)	24.95 (7.55)	F = 0.078 *p* = 0.925
	Post	26.24 (9.06)	25.75 (9.18)	25.39 (8.20)	

Means and standard deviations between brackets. Only significant interaction is reported

**Fig. 1:**
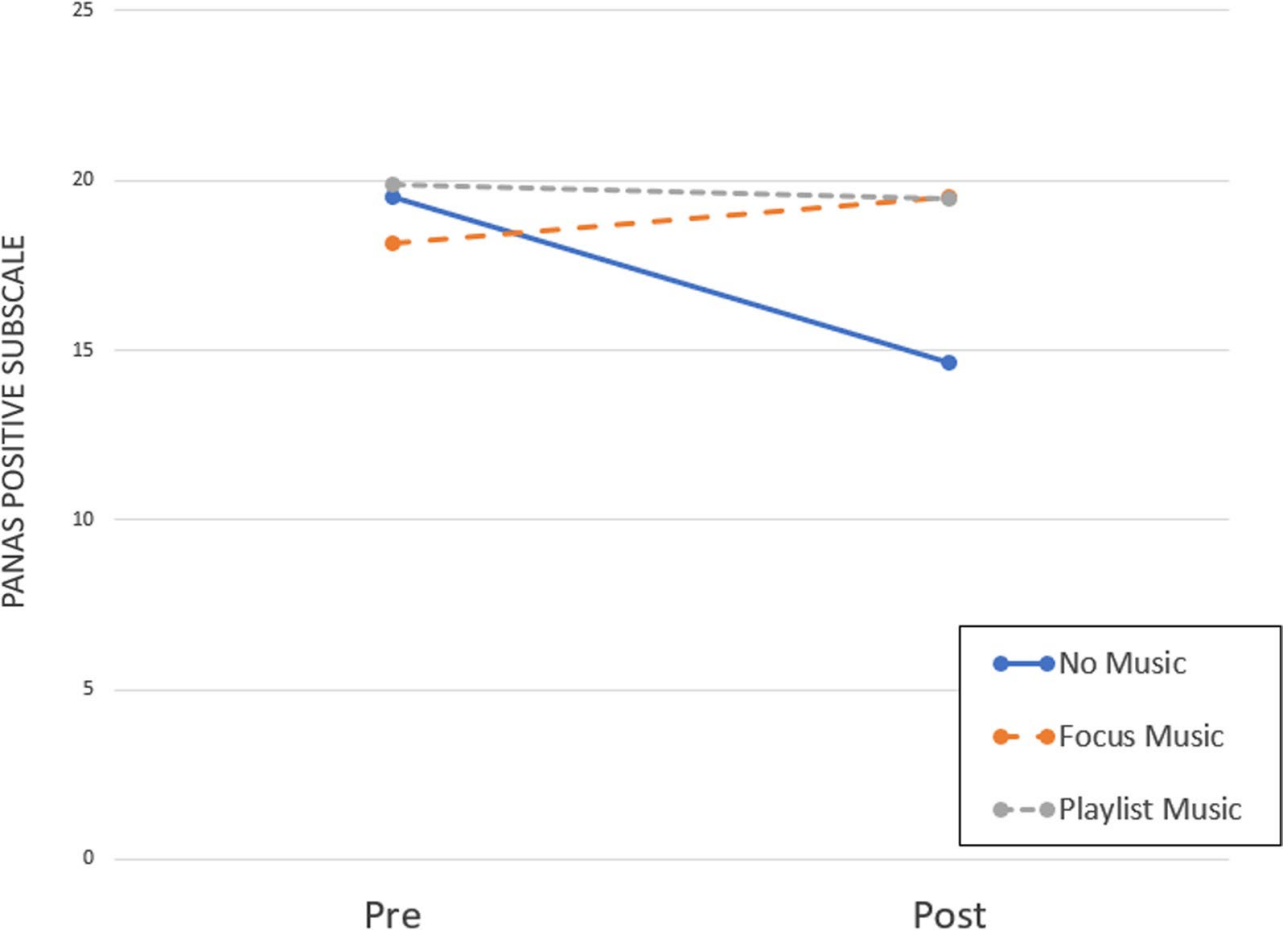
Graphical representation of distribution of the PANAS positive subscale scores before and after mealtime under the three different music conditions. The interaction time by condition was significant and the only condition that showed a significant reduction in scores between times was the no music condition (*Z* = −4.858, *p* < 0.001)

Repeated measure ANOVA conducted on hunger revealed a significant reduction over time (F(1,150) = 97.447, *p* < 0.001, η_p_^2^ = 0.394), but a nonsignificant effect of the type of music (F (2,150) = 0.033, *p* = 0.968) and a nonsignificant interaction time by type of music (F (2,150) = 0.189, *p* = 0.828). The same was found for satiety, with a significant increase over time (F(1,150) = 255.630, *p* < 0.001, η_p_^2^ = 0.630), but a nonsignificant effect of the type of music (F (2,150) = 0.036, p = 0.965), and a nonsignificant interaction time by the type of music (F (2,150) = 0.129, *p* = 0.879). Looking at the desire to eat, the same results were found with a significant reduction over time (F(1,150) = 104.415, *p* < 0.001, η_p_^2^ = 0.410), but a nonsignificant effect of music type (F(2,150) = 0.339, *p* = 0.713) and a non-significant interaction time by type of music (F (2,150) = 0.411, *p* = 0.664). The same results were found for the stress level, with a significant effect of time (F(1,150) = 183.471, *p* < 0.001, η_p_^2^ = 0.550), but a nonsignificant effect of the type of music (F (2,150) = 0.033, *p* = 0.967), and a nonsignificant interaction time by the type of music (F (2,150) = 0.326, *p* = 0.722). Looking at perceived negative emotions, no time (F(1,150) = 0.239, p = 0.626) or music (F(2,150) = 0.384, *p* = 0.682) were found; thus, no results for the time by music (F (2,150) = 0.078, *p* = 0.925). Finally, looking at perceived positive emotions, we found a significant effect of time (F(1,150) = 27.250, *p* < 0.001, η_p_^2^ = 0.154), a significant effect of the musical conditions (F(2,150) = 3.173, *p* = 0.045, η_p_^2^ = 0.041), and a significant interaction time by music (F(2,150) = 26.920, *p* < 0.001, η_p_^2^ = 0.264). Looking at pairwise comparisons between music conditions, a significant difference was found between focus music and no music (*p* = 0.046) and playlist and no music (*p* = 0.022), but no differences were found between playlist and focus music (*p* = 0.762). See Fig. 1 for a graphical representation.

## Discussion

This study adopts an ecological approach, pioneering the examination of background music’s impact on individuals with ED during mealtime in an inpatient facility, considering various music choices. Our findings reveal a positive influence of background music during meals, particularly in reducing negative emotional states. This effect was most pronounced in the absence of background music. Although we did not observe significant differences between the types of music, the general benefit of music during mealtime was evident.

Recent literature has underscored the importance of incorporating music into treatment protocols, although consensus remains elusive due to methodological variations between studies [44]. Previous research, which included classical music during mealtime, demonstrated positive results, particularly among individuals with anorexia nervosa and bulimia nervosa [23, 24]. Our findings align with this evidence, highlighting the positive impact of background music across the entire spectrum of eating disorders. Participants reported better emotional states and averted post-meal mood deterioration, implying the potential utility of integrating background music into the ED treatment settings during mealtime, as previously identified [12]. However, even though music demonstrated a potential effect in preventing the deterioration of positive moods, we need to assess the absence of other specific effects in our study. Despite literature reporting an active role of music in emotional regulation and stress reduction, its application during mealtime appears to be less effective [45–47]. This discrepancy could be attributed to the collection of data close to the end of mealtime, or it might be influenced by the specific context of a particularly challenging moment, where patients could face difficulty in selective attention [45, 48]. While our study is unable to clarify this point, future studies should evaluate these aspects.

The ability of music to alleviate negative moods is well established, and music interventions are commonly employed to mitigate negative emotions in various settings, offering both psychological and physiological benefits [27, 49]. Furthermore, existing literature suggests that the style of music does not exert significant moderating effects on mood during mealtime [27], reinforcing the role of music—in general—as a potent distraction from food-related concerns. Our findings, which revealed reduced uneaten food when music was present, are consistent with observations in the general population [50, 51]. Overall, our data show the presence of an improvement in mealtime outcomes using background music in an inpatient setting.

When examining uneaten foods, an interesting observation emerged: a decrease in protein and lipid intake was noted in the silent scenario. In the nutritional rehabilitation of people with ED, an appropriate amount of fat and protein is crucial for several reasons: nutrient density, energy source, muscle maintenance, hormonal balance, satiety, and brain function [52, 53]. Indeed, this finding is intriguing because protein is essential for the synthesis of serotonin and dopamine, neurotransmitters that play a pivotal role in fostering feelings of positivity, motivation, passion, tranquility, and presence [54]. Similarly, lipids are crucial for neural development, nerve cell differentiation, and migration, making them vitally important for the proper functioning of the nervous system and for activating reward-related areas in the brain [55, 56]. Therefore, the decline in mood may be exacerbated also by the reduction in specific dietary intake. The presence of music might help mitigate these effects, assisting people in improving their dietary quality [51]. However, this aspect should be assessed in future studies to explore its potential effects on eating rehabilitation.

Finally, a particularly intriguing result emerged, showing reduced eating rituals documented during mealtime when background music was introduced. Psychological and neurobiological data indicate that eating rituals in EDs are associated with obsessive–compulsive traits and negative responses to stressors, which can hinder progress and treatment effectiveness [57, 58]. This underscores the need for studies to improve results in this regard. Few studies have explored the impact of exposure to music on obsessive–compulsive symptoms, with limited data suggesting a positive role of music in this specific psychological disorder [59]. Our findings imply that external elements, such as music, can help patients shift their focus away from negative emotional states that could be linked to rumination and disruptive thoughts [59, 60]. Additionally, our results support the potential of music in reducing the degradation of positive emotions, although more research is warranted to dive into this area.

### Limitations, strengths, and future directions

One of the notable limitations of this study is the predominance of participants diagnosed with anorexia nervosa, with fewer individuals diagnosed with bulimia nervosa or binge eating disorder. To mitigate the variation between individuals, we employed a randomized repetition of exposure to music under all conditions. A notable strength of this study lies in its application of diverse conditions within real-world settings, demonstrating the potential practicality of the results in inpatient facilities. However, for a broader generalizability, it may be advisable to replicate the study with outpatient populations. Additionally, we relied exclusively on self-report questionnaires to assess affective changes and there is a reduced heterogeneity of the sample. Future research could improve this aspect by incorporating a variety of methodologies and different populations (i.e., men and gender-diverse individuals) to ensure a more comprehensive evaluation. Additionally, for future studies, we acknowledge the potential for exploring mediation effects, particularly investigating whether the observed effects of music on mealtime benefits are mediated by increases in mood and decreases in anxiety. The examination of this mediation pathway, from music to mood and subsequently to mealtime benefits, could offer valuable insights and contribute to a deeper understanding of the underlying mechanisms involved.

## Conclusion

In summary, the findings affirm the role of music as a beneficial environmental distractor during mealtime for people with ED. The introduction of background music may enhance the overall inpatient treatment experience, creating a more supportive and accommodating atmosphere, particularly during stressful moments like mealtime. This approach has the potential to be considered for implementation in all ED inpatient facilities, with the aim of promoting positive affective states that could, in turn, produce positive effects on both psychological and physical symptoms during meals, ultimately contributing to improved overall outcomes.

## Data Availability

The research team will hold the anonymized data. Data sharing will be considered for researchers who provide a methodologically sound proposal.

## References

[1] Ágh T, Kovács G, Supina D, Pawaskar M, Herman BK, Vokó Z (2016). A systematic review of the health-related quality of life and economic burdens of anorexia nervosa, bulimia nervosa, and binge eating disorder. Eat Weight Disorders-Stud Anorexia Bulimia Obesity.

[2] Hetherington MM, Rolls BJ (2001). Dysfunctional eating in the eating disorders. Psychiatr Clin North Am.

[3] Meneguzzo P, Todisco P, Calonaci S, Mancini C, Dal Brun D, Collantoni E, et al. Health-related quality of life assessment in eating disorders: adjustment and validation of a specific scale with the inclusion of an interpersonal domain. Eating and Weight Disorders. 2020. 10.1007/s40519-020-01081-5 PMC8437832 33315213

[4] Holmes M, Fuller-Tyszkiewicz M, Skouteris H, Broadbent J (2015). Understanding the link between body image and binge eating: a model comparison approach. Eat Weight Disord.

[5] Rieder S, Ruderman A (2001). Cognitive factors associated with binge and purge eating behaviors: the interaction of body dissatisfaction and body image importance. Cognit Ther Res.

[6] Vervaet M, Puttevils L, Hoekstra RHA, Fried E, Vanderhasselt MA (2021). Transdiagnostic vulnerability factors in eating disorders: a network analysis. Eur Eat Disord Rev.

[7] Solmi M, Gallicchio D, Collantoni E, Meneguzzo P, Zanetti T, Degortes D (2018). The impact of weight suppression and weight loss speed on baseline clinical characteristics and response to treatment. Int J Eat Disord.

[8] Hart S, Abraham S, Luscombe G, Russell J (2008). Eating disorder management in hospital patients: current practice among dietitians in Australia. Nutr Diet.

[9] Hay P (2020). Current approach to eating disorders: a clinical update. Intern Med J.

[10] Long S, Wallis DJ, Leung N, Arcelus J, Meyer C. Mealtimes on eating disorder wards: A two-study investigation. International J Eat Disorders. 2012. p. 241–6. 10.1002/eat.20916 21472761

[11] Offord A, Turner H, Cooper M (2006). Adolescent inpatient treatment for anorexia nervosa: a qualitative study exploring young adults’ retrospective views of treatment and discharge. Eur Eat Disord Rev.

[12] Hage TW, Rø Ø, Moen A. “ Time’s up”—Staff’s management of mealtimes on inpatient eating disorder units. J Eat Disord. 2015;3. 10.1186/s40337-015-0052-4 PMC4389322 25861449

[13] Long S, Wallis D, Leung N, Meyer C. “All eyes are on you”: Anorexia nervosa patient perspectives of in-patient mealtimes. J Health Psychol. 2012. p. 419–28. 10.1177/1359105311419270 21868423

[14] Gilon Mann T, Hamdan S, Bar-Haim Y, Lazarov A, Enoch-Levy A, Dubnov-Raz G (2018). Different attention bias patterns in anorexia nervosa restricting and binge/purge types. Eur Eat Disord Rev.

[15] Halbeisen G, Brandt G, Paslakis G. A plea for diversity in eating disorders research. Front Psychiatry. 2022;13. 10.3389/fpsyt.2022.820043 PMC8894317 35250670

[16] Lacalaprice D, Mocini E, Frigerio F, Minnetti M, Piciocchi C, Donini LM, et al. Effects of mealtime assistance in the nutritional rehabilitation of eating disorders. Eating and Weight Disorders. Springer Science and Business Media Deutschland GmbH; 2023. 10.1007/s40519-023-01605-9 PMC10492868 37688675

[17] Elran-Barak R, Sztainer M, Goldschmidt AB, Crow SJ, Peterson CB, Hill LL (2015). Dietary restriction behaviors and binge eating in Anorexia Nervosa, Bulimia Nervosa and binge eating disorder: trans-diagnostic examination of the restraint model. Eat Behav.

[18] Kells M, Davidson K, Hitchko L, O’Neil K, Schubert-Bob P, McCabe M (2013). Examining supervised meals in patients with restrictive eating disorders. Appl Nurs Res.

[19] Kells M, Schubert-Bob P, Nagle K, Hitchko L, O’Neil K, Forbes P (2017). Meal supervision during medical hospitalization for eating disorders. Clin Nurs Res.

[20] Anderson LM, Crow SJ, Peterson CB (2014). The impact of meal consumption on emotion among individuals with eating disorders. Eat Weight Disord.

[21] Harvey T, Troop NA, Treasure JL, Murphy T (2002). Fear, disgust, and abnormal eating attitudes: a preliminary study. Int J Eat Disord.

[22] Watts LM. Meal support therapy, Part 1. Eating Disorders Review for Professionals. 2000;11.

[23] Cardi V, Kan C, Roncero M, Harrison A, Lounes N, Tchanturia K, et al. Mealtime support in anorexia nervosa: a within-subject comparison study of a novel vodcast intervention. Psychother Psychosom. 2011. p. 54–5. 10.1159/000329992 22123183

[24] Cardi V, Esposito M, Clarke A, Schifano S, Treasure J. The impact of induced positive mood on symptomatic behaviour in eating disorders: an experimental, AB/BA crossover design testing a multimodal presentation during a test-meal. Appetite. 2015;87:192–8. 10.1016/j.appet.2014.12.224 25555537

[25] Ryan V, Malson H, Clarke S, Anderson G, Kohn M (2006). Discursive constructions of “eating disorders nursing”: an analysis of nurses’ accounts of nursing eating disorder patients. Eur Eat Disord Rev.

[26] Fachner J. Music, Moments, and healing process: music therapy. The Routledge Companion to Music Cognition. Routledge; 2017. p. 89–99.

[27] de Witte M, Spruit A, van Hooren S, Moonen X, Stams GJ (2020). Effects of music interventions on stress-related outcomes: a systematic review and two meta-analyses. Health Psychol Rev.

[28] Thoma MV, Zemp M, Kreienbühl L, Hofer D, Schmidlin PR, Attin T (2015). Effects of music listening on pre-treatment anxiety and stress levels in a dental hygiene recall population. Int J Behav Med.

[29] Harada T, Kurai R, Ito S, Nitta Y, Aoi S, Ikeda H (2017). Effect of joyful and anxiety-provoking music on autonomic nervous system function. Int Med J.

[30] Bibb J, Castle D, Skewes MK (2019). Reducing anxiety through music therapy at an outpatient eating disorder recovery service. J Creat Ment Health.

[31] Milliard RE. The use of cognitive-behavioral music therapy in the treatment of women with eating disorders. Music Ther Perspect. 2001;19:109–13. http://mtp.oxfordjournals.org/

[32] Pasiali V, Quick D, Hassall J, Park HA. Music therapy programming for persons with eating disorders. Voices: A World Forum for Music Therapy. 2020;20:15.

[33] Groarke JM, Groarke AM, Hogan MJ, Costello L, Lynch D (2020). Does listening to music regulate negative affect in a stressful situation? Examining the effects of self-selected and researcher-selected music using both silent and active controls. Appl Psychol Health Well Being.

[34] Cook T, Roy ARK, Welker KM (2019). Music as an emotion regulation strategy: an examination of genres of music and their roles in emotion regulation. Psychol Music.

[35] Rentfrow PJ, Gosling SD (2003). The Do Re Mi’s of Everyday Life: the structure and personality correlates of music preferences. J Pers Soc Psychol.

[36] Iwanaga M, Moroki Y. Subjective and Physiological Responses to Music Stimuli Controlled Over Activity and Preference Downloaded from [Internet]. Journal of Music Therapy, XXXVI. 1999. Available from: http://jmt.oxfordjournals.org/10.1093/jmt/36.1.26 10519843

[37] Todisco P, Meneguzzo P, Garolla A, Antoniades A, Vogazianos P, Tozzi F. Impulsive behaviors and clinical outcomes following a flexible intensive inpatient treatment for eating disorders: findings from an observational study. Eating and Weight Disorders. 2020; 10.1007/s40519-020-00916-5 32430886

[38] American Psychiatric Association. Diagnostic and statistical manual of mental disorders (DSM-5). American Psychiatric Association. Washington, DC. Author.; 2013.

[39] Luce KH, Crowther JH (1999). The reliability of the Eating Disorder Examination - Self-Report Questionnaire Version (EDE-Q). Int J Eat Disord.

[40] Watson D, Clark LA, Tellegen A (1988). Development and validation of brief measures of positive and negative affect: the PANAS scales. J Pers Soc Psychol.

[41] Calugi S, Chignola E, Grave RD. A longitudinal study of eating rituals in patients with anorexia nervosa. Front Psychol. 2019;10. 10.3389/fpsyg.2019.00015 PMC6345676 30713513

[42] Marletta L, Carnovale E. Tabelle di composizione degli alimenti. Milano: Istituto Nazionale di Ricerca per gli Alimenti e la Nutrizione. 2000;

[43] Mamalaki E, Zachari K, Karfopoulou E, Zervas E, Yannakoulia M. Presence of music while eating: Effects on energy intake, eating rate and appetite sensations. Physiol Behav [Internet]. 2017;168:31–3. Available from: 10.1016/j.physbeh.2016.10.019 10.1016/j.physbeh.2016.10.019 27789251

[44] Testa F, Arunachalam S, Heiderscheit A, Himmerich H (2021). A systematic review of scientific studies on the effects of music in people with or at risk for eating disorders. Psychiatr Danub.

[45] Spalatro AV, Marzolla M, Vighetti S, Daga GA, Fassino S, Vitiello B (2021). The song of Anorexia Nervosa: a specific evoked potential response to musical stimuli in affected participants. Eat Weight Disord.

[46] van den Tol AJM, Coulthard H, Hanser WE (2020). Music listening as a potential aid in reducing emotional eating: an exploratory study. Music Sci.

[47] Krishna Priya A, Applewhite B, Au K, Oyeleye O, Walton E, Norton C, et al. Attitudes surrounding music of patients with anorexia nervosa: a survey-based mixed-methods analysis. Front Psychiatry. 2021;12. 10.3389/fpsyt.2021.639202 PMC8206484 34149472

[48] Ceccato E, Roveran C. Effects of music therapy in the reduction of pre-meal anxiety in patients suffering from anorexia nervosa. Brain Sci. 2022;12. 10.3390/brainsci12060801 PMC9221392 35741686

[49] de Witte M, Pinho A da S, Stams G-J, Moonen X, Bos AER, van Hooren S. Music therapy for stress reduction: a systematic review and meta-analysis. Health Psychol Rev. 2022;16:134–59. 10.1080/17437199.2020.1846580 33176590

[50] Cui T, Xi J, Tang C, Song J, He J, Brytek-Matera A (2021). the relationship between music and food intake: a systematic review and meta-analysis. Nutrients.

[51] Stroebele N, de Castro JM (2006). Listening to music while eating is related to increases in people’s food intake and meal duration. Appetite.

[52] Hart S, Marnane C, McMaster C, Thomas A. Development of the “Recovery from Eating Disorders for Life” Food Guide (REAL Food Guide): a food pyramid for adults with an eating disorder. J Eat Disord. 2018;6. 10.1186/s40337-018-0192-4 PMC5878939 29619220

[53] Reiter CS, Graves L. Nutrition therapy for eating disorders. Nutr Clin Pract. 2010. p. 122–36. 10.1177/0884533610361606 20413693

[54] Strasser B, Gostner JM, Fuchs D (2016). Mood, food, and cognition: role of tryptophan and serotonin. Curr Opin Clin Nutr Metab Care.

[55] AlAmmar WA, Albeesh FH, Khattab RY. Food and Mood: the Corresponsive Effect. Curr Nutr Rep. Springer; 2020. p. 296–308. 10.1007/s13668-020-00331-3 32623655

[56] Gibson EL. Mood, emotions and food choice. The psychology of food choice. CABI Wallingford UK; 2006. p. 113–40.

[57] Steinglass J, Walsh BT (2006). Habit learning and anorexia nervosa: a cognitive neuroscience hypothesis. Int J Eat Disord.

[58] Sunday SR, Halmi KA (2000). Comparison of the Yale-Brown-Cornell Eating Disorders Scale in recovered eating disorder restrained dieters, and nondieting controls. Int J Eat Disord.

[59] Truong TPA, Applewhite B, Heiderscheit A, Himmerich H (2021). A systematic review of scientific studies and case reports on music and obsessive-compulsive disorder. Int J Environ Res Public Health.

[60] Bidabadi SS, Mehryar A (2015). Music therapy as an adjunct to standard treatment for obsessive compulsive disorder and co-morbid anxiety and depression: a randomized clinical trial. J Affect Disord.

